# Mechanisms of Body Weight Reduction by Black Tea Polyphenols

**DOI:** 10.3390/molecules21121659

**Published:** 2016-12-07

**Authors:** Haibo Pan, Ying Gao, Youying Tu

**Affiliations:** Tea Research Institute of Zhejiang University, Hangzhou 310058, China; apanhaibo@126.com (H.P.); Yinggao6023@gmail.com (Y.G.)

**Keywords:** anti-obesity, black tea polyphenol, lipid digestion, saccharide digestion, AMPK, anti-oxidation

## Abstract

Obesity is one of the most common nutritional diseases worldwide. This disease causes health problems, such as dyslipidemia, hyperglycemia, hypertension and inflammation. There are drugs used to inhibit obesity. However, they have serious side effects outweighing their beneficial effects. Black tea, commonly referred to as “fermented tea”, has shown a positive effect on reducing body weight in animal models. Black tea polyphenols are the major components in black tea which reduce body weight. Black tea polyphenols are more effective than green tea polyphenols. Black tea polyphenols exert a positive effect on inhibiting obesity involving in two major mechanisms: (i) inhibiting lipid and saccharide digestion, absorption and intake, thus reducing calorie intake; and (ii) promoting lipid metabolism by activating AMP-activated protein kinase to attenuate lipogenesis and enhance lipolysis, and decreasing lipid accumulation by inhibiting the differentiation and proliferation of preadipocytes; (iii) blocking the pathological processes of obesity and comorbidities of obesity by reducing oxidative stress. Epidemiological studies of the health relevance between anti-obesity and black tea polyphenols consumption remain to be further investigated.

## 1. Introduction

Obesity is one of the most common nutritional diseases worldwide. According to the World Health Organization (WHO) 2010 global report on non-communicable diseases, 2.8 million lives are claimed by overweight/obesity annually [1]. WHO defines obesity as a body mass index (BMI) equal to or greater than 30 kg/m^2^. Obesity leads to serious illnesses, such as cardiovascular diseases, type 2 diabetes, and cancer [2,3,4]. Physiologically, obesity arises from a metabolic imbalance in the organs and tissues of the human body between energy expenditure and energy intake which, in turn, leads to increasing fat accumulation in adipose tissue [5]. Treatment in obese/overweight individuals has been centered on helping them curb their weight through a calorie-restricted diet and increased physical activity.

In the long term, restricting calories may not be effective biologically or behaviorally. Therefore, drugs have been used to treat obesity. There are two different drugs to treat obesity currently on the market. Orlistat inhibits pancreatic lipase and reduces intestinal fat absorption. Sibutramine is an anorectic or appetite suppressant. Both drugs have unwanted side effects, such as increased blood pressure, astriction, headache, and insomnia [6]. For this reason, black tea has been explored for its obesity treatment potential.

Tea is one of the most popular beverages worldwide and is second only to water in popularity. Megatons of tea are produced worldwide annually. Different manufacturing processes produce different kinds of tea. Tea is generally divided into three types: non-fermented green tea, semi-fermented oolong tea, and fermented black tea. The major type of tea produced and consumed worldwide is black tea. Tea has been used as a drug since antiquity. Many studies have shown that tea polyphenols are the major effective components in teas, e.g., used for its anti-oxidation [7,8,9], anti-carcinoma [10,11,12], and arteriosclerosis prevention [13] properties, and in the prevention of Alzheimer’s and Parkinson diseases [14,15,16]. Over the past decades, black tea polyphenols have been studied for their anti-obesity effects. There is increasing evidence to demonstrate that black tea polyphenols have an effect on preventing obesity. Numerous studies have reported the anti-obesity effect of black tea polyphenols in animals [17,18,19] and humans [20,21]. However, there is no systematic review of the literature summarizing the association between black tea polyphenols and obesity. The present article reviews the evidence and discusses the molecular mechanisms for the anti-obesity effect of black tea polyphenols. It is hoped that this article will enhance our understanding of the anti-obesity effects of black tea consumption and stimulate future research.

## 2. The Effect of Black Tea Polyphenols on Lipid Digestion, Absorption and Intake

Fat digestion is an interfacial process which depends on the adsorption of lipase on the surface of lipid droplets. The formation of emulsion droplets provides a surface for lipase adsorption which influences the anchoring of lipases, and plays an important role in the rate of lipid digestion. In a model system simulating small intestinal conditions, it was observed that the initial droplet size changed from 1.4 to 25.9 μm in an emulsion of 0.10 mg/mL black tea extract. Black tea extract inhibited the emulsion of droplets and reduced the surface area. Besides, black tea extract was more effective in changing the emulsion properties than green tea extract due to the thearubigins and theaflavins in black tea [22]. It was also found that theaflavins inhibited the incorporation of 14C-labeled cholesterol into simulative dietary mixed micelles and increased their particle size. These particles were composed of insoluble, large multilamellar vesicles with onion-like structures in the presence of theaflavins [23].

Adsorption of lipase on the surface of emulsified fat droplets causes a conformational rearrangement, and the enzymatic hydrolysis of triglycerides (TGs) by specific lipases occurs. Black tea polyphenols dose-dependently inhibited the activity of pancreatic lipase in vitro with an IC_50_ of 0.254 mg/mL. Theaflavins with galloyl moieties inhibited the activity of pancreatic lipase in vitro with an IC_50_ of about 0.5 mg/mL. Theaflavins with galloyl moieties were more potent in inactivating pancreatic lipase than theaflavin (TF1) which got a similar inhibiting effect to epigallocatechin gallate (EGCG), epicatechin gallate (ECG), and a mixture of EGCG and ECG [18]. However, as reported by Nakai, Masaaki et al., a theaflavin was more effective in suppressing the activity of pancreatic lipase than EGCG, and theaflavins suppressed the activity of pancreatic lipase in vitro with a similar IC_50_ [24]. It was also reported that black tea extract and black tea polyphenols from black tea extract inhibited pancreatic lipase activity with an IC_50_ of 15.5 and 36.4 mg/mL in vitro (5). Black tea polyphenols were the major active components of black tea extract that inhibited pancreatic lipase, as also noted by Shishikura [22] and Hamdaoui [25].

The hydrolysis products of fat will be absorbed into plasma through the intestine. As the activity of lipase is inhibited by black tea polyphenols, much fewer hydrolysis products of fat will be absorbed into the plasma. Black tea polyphenols significantly alleviated the increase in serum glucose, total lipid, TG and phospholipid levels in rat models [26,27]. Anti-obesity and lipid-lowering effects of theaflavins with high purity on high-fat diet–induced obese rats were investigated [19,28]. The body weight of obese rats was significantly decreased by TF1. TF1 remarkably decreased the adiposity index and the serum levels of TG and low-density lipoprotein cholesterol (LDL-C). A highly purified theaflavin mixture (TFs, 83.84%) remarkably decreased the adiposity index and the serum levels of total cholesterol (TC), TG and LDL-C. It was also found that TF1 remarkably decreased the food intake of obese rats, which was consistent with the results of Yatao Du [29]. However, other reports indicate that black tea decreased body weight without altering the food intake [27,30]. The black tea polyphenols could increase the fecal fatty acid content, thereby preventing high-fat diet–induced obesity [17]. The effect of black tea extract on lipid excretion was also found in a randomized, placebo-controlled, double-blind crossover study. Total lipid excretion increased after black tea extract intake in comparison with the control which indicated that black tea extract increased lipid excretion [20]. The black tea extract used in the study contained 5% black tea polyphenols. It is unclear whether the components of black tea contributed to the lipid excretion. To confirm exactly which components of tea are responsible for lipid excretion, both a comparison of the effects of green and black tea and an examination of the effects of individual components of black tea may be helpful.

## 3. The Effect of Black Tea Polyphenols on Lipid Metabolism and Accumulation

After being absorbed through the intestines, lipids are transported to liver cells, muscle cells or adipocytes through the lymph system. Lipids are mainly sent to liver cells, muscle cells and adipocytes. The lipids sent to liver cells are esterified to triglycerides, converted into very-low-density lipoprotein (VLDL), and released into the blood to transport endogenous-derived lipids. Lipids sent to muscle cells can be oxidized in the mitochondria for energy. Lipids sent to fat cells are stored until they are needed for energy at a later time.

Fat accumulation is controlled by the balance between lipogenesis and lipolysis [31]. Lipogenesis is a metabolic process that converts simple sugars to fatty acids and synthesizes triglycerides through the reaction of fatty acids with glycerol. Lipolysis is the process in which triglycerides are hydrolyzed to generate glycerol and free fatty acids. Lipogenesis takes place and converts the excess energy into body fat. Otherwise, lipolysis happens when energy expenditure is in excess of energy intake.

Insulin, a hormone secreted from the pancreas, is involved in the regulation of lipogenesis. Theaflavins are identified as novel mimics of insulin/IGF-1, acting on the phosphorylation of mammalian forkhead transcription factor family O1a (FOXO1a) and subsequently on the inactivation of the promoter of the phosphoenolpyruvate carboxykinase (PEPCK) gene [32]. PEPCK is an important enzyme in hepatic gluconeogenesis. In this way, theaflavins suppress gluconeogenesis.

Monocyte Chemoattractant Protein-1 (MCP-1), the most extensively studied CC chemokine, has a direct role in the development of obesity. The plasma level of MCP-1 was found increased in obese patients [33,34] and obese mice [35]. It was correlated with the number and volume of omental adipocytes [36] and the area of visceral adiposity [37] in humans. Black tea polyphenols significantly lowered the body weight, total visceral fat volume, and liver lipid weight in mice fed high-fat, high-sucrose obesogenic diets [38]. In mesenteric fat and liver tissue, Mcp1 gene expression was significantly decreased by black tea polyphenols.

Fatty acid synthase (FAS) regulates the rate-limiting step in fatty acid synthesis. Mammalian FAS is a homodimer which consists of four domains (including ketoacyl reductase) in the C-terminal section and three domains in the N-terminal section. Theaflavins inhibit the activity and expression of FAS. Theaflavins are competitive inhibitors of NADPH (a substrate of b-ketoacyl reductase of type I and II FAS), indicating that theaflavins may compete with NADPH for the same binding site [39]. The expression of FAS is significantly suppressed by theaflavin monomers TF1, theaflavin 3-gallate (TF2a), theaflavin 3′-gallate (TF2b), and theaflavin 3,3′-digallate (TF3) at both protein and mRNA levels [40]. The underlying mechanism is that theaflavins down-regulate the EGF receptor/PI3K/Akt/Sp-1 signal transduction pathways. By targeting this pathway, TF3 significantly reduces EGF-induced biosynthesis of triglycerides, cholesterol and fatty acids.

AMP-activated protein kinase (AMPK) is an important therapeutic target for obesity treatment [41] and a major target of theaflavins for their anti-obesity effect. AMPK plays a central role in regulating cellular and organismal metabolism in eukaryotes [42]. It enhances hepatic fatty acid oxidation and ketogenesis, and attenuates cholesterol synthesis, fatty acid synthesis and triglyceride synthesis [43].

AMPK is an upstream protein for the metabolic enzymes Acetyl-CoA carboxylase and HMG-CoA reductase, which mediate the rate-limiting steps for fatty acid and sterol synthesis, respectively [42]. Lin et al. proved that theaflavins significantly decreased lipid accumulation, reduced fatty acid synthesis, and promoted fatty acid oxidation in vitro and in vivo. One of the mechanisms was that theaflavins inhibited Acetyl-CoA carboxylase activities by stimulating AMPK through the liver kinase B1 and reactive oxygen species pathways [44]. Singh et al. demonstrated that black tea decreased cholesterol synthesis by directly suppressing HMG-CoA reductase and by stimulating its inactivation through phosphorylating AMPK [45].

The activation of AMPK promotes fatty acid oxidation and increases lipolysis. A single oral administration of theaflavins led to increased energy expenditure, along with an increase in uncoupling protein (UCP) and peroxisome proliferator-activated receptor gamma coactivator-1α (PGC-1α) mRNA expression and phosphor-AMPKα in brown adipose tissue and the gastrocnemius muscle in mice [46]. UCP and PGC-1α are partially responsible for adaptive thermogenesis and lipolysis. Black tea suppresses adiposity via phosphorylation of AMPK and increases UCP-1 expression as a marker for the conversion of white to brown adipose tissue [47]. Similarly, a recent study showed that TF3 activates AMPK to up-regulate peroxisome proliferator-activated receptor α (PPARα) [48]. PPARα is a transactivator for the expression of lipolytic genes, such as mitochondrial UCPs. Increased PPARα then promotes the expression of genes which encode proteins that favor lipolysis, β-oxidation or energy dissipation, resulting in a decreased triacylglycerol level in TF3-treated adipocytes. Moreover, the activation of AMPK by TF3 reverses the inactivation of Forkhead box O3A (FoxO3A) and insulin-induced suppression of manganese superoxide dismutase (MnSOD) in adipocytes. FoxO3A is a common target transcription factor for AMPK signaling and mediates the cellular response against oxidative stress by up-regulation of MnSOD promoter activity. Down-regulation of MnSOD progresses atherosclerosis and diabetic nephropathy [48].

Black tea polyphenols act on the nuclear receptors, in addition to their activation of AMPK, to influence glucose and fatty acid metabolism. Nuclear receptors are sensors for steroid hormones, thyroid hormones and some specific molecules. Peroxisome proliferator-activated receptors (PPARs), liver X receptors (LXRs), the Farnesoid X receptor (FXR) and retinoid X receptors (RXRs) belong to the nuclear receptor superfamily. Activation of PPARγ leads to insulin sensitization and stimulates glucose metabolism, while activation of PPARβ/δ stimulates fatty acid metabolism [49]. LXRs protect the cells from cholesterol over-accumulation by promoting reverse cholesterol transport and cholesterol conversion to bile acids in the liver [50]. FXR controls the lipid and glucose metabolism, as well as the synthesis, conjugation and transport of bile acid [51]. The RXR heterodimerizes with many other nuclear receptors (e.g., FXR, LXR, and PPAR) to regulate metabolism-related gene expressions [52]. Using high-throughput screening technology, Wang proved that theaflavins showed slight activation of PPARδ, PPARγ, LXR and FXR [53]. Deng et al. demonstrated that black tea polyphenols induced the gene expression of RXRa, RXRb and NR1D1 [54]. It indicated that these nuclear receptors might be responsible for the anti-obesity effect of theaflavins.

In addition, black tea polyphenols affect the gene expression of low-density lipoprotein receptor (LDLR) and acetyl-CoA acetyltransferase 1 (ACAT1) [54]. LDLR mediates the endocytosis of cholesterol-rich LDL. ACAT1 catalyzes the reversible formation of acetoacetyl-CoA from acetyl-CoA.

When the generation of lipid exceeds the consumption, spare lipids are stored in lipid droplets. Over-accumulation of lipids in adipocytes results in the increase of the size and number of adipocytes, leading to obesity and related diseases. Theaflavins have been reported to suppress the adipogenic differentiation of stem cells, the differentiation of preadipocytes to adipocytes and the proliferation of preadipocytes. Theaflavins inhibited the differentiation of rabbit bone marrow mesenchymal stem cells into adipocytes [55]. Compared with the control group, the differentiation efficiency in the theaflavins-treated group was reduced by half. Sun et al. have demonstrated that theaflavins were able to inhibit the proliferation and differentiation of 3T3-L1 preadipocytes and decreased the intracellular content of triglycerides [56]. Compared with tea polyphenols, the activity of theaflavins was much stronger. Gene expression profile analysis revealed that black tea polyphenols down-regulated the gene expression of adipose differentiation-related protein (ADRP). ADRP is expressed early during adipose differentiation and stimulates lipid accumulation and lipid droplet formation [57]. An animal study confirmed the effectiveness of theaflavins in vivo. Black tea polyphenols prevented the increase in the body weight, peritoneal and epididymal adipocyte tissue weight, liver weight, plasma total cholesterol level, plasma total low-density lipoprotein (LDL) level and plasma triglycerides level in high-fat diet–fed rats [58]. Black tea polyphenols decreased the mRNA level of three adipocyte-specific genes (adipocyte acid-binding protein 2, tumor necrosis factor-alpha and leptin) in the adipocyte tissue of high-fat diet–fed rats, suggesting an inhibition of the adipogenesis [58].

Taken together, theaflavins interfere with multiple processes in lipid synthesis, metabolism, transport and storage to exhibit anti-obesity activities, suggesting their potential in obesity treatment.

## 4. The Effect of Black Tea Polyphenols on Saccharide Digestion and Absorption

The inhibitory activities of black tea polyphenols against α-amylase and glucosidases as well as glucose transporters have been demonstrated in many studies. The inhibition of α-amylase from human saliva by polyphenols of tea was investigated in vitro. Theaflavins showed stronger inhibitory effect than catechins [59]. Polymer polyphenols of black tea isolated with solvents showed comparable inhibitory activities of amylase. Polymer polyphenols in ethyl acetate and n-butyl alcohol showed inhibitory activities of 47.7% and 46.8%, respectively. Polymer polyphenols showed a stronger inhibitory effect on amylase than on theaflavins and EGCG [60]. Esterified polyphenols were potent inhibitors of the sucrose and α-glucosidase activity in the rat small intestine [61]. In another study, the inhibitory effects of theaflavins against α-glucosidase were observed [62]. TF2a suppressed glucose production from maltose through the inhibition of α-glucosidase in the gut. Black tea polyphenols were separated by C18 and LH-20 extraction into a hydrophobic fraction, a low-molecular-weight phenolic-enriched fraction, and a high-molecular-weight enriched fraction. The high-molecular-weight enriched fraction was the most bioactive against a-glucosidase, followed by the hydrophobic fraction [63]. Black tea extracts had the highest α-glucosidase inhibitory activity, followed by white tea, oolong tea and green tea [64]. It was demonstrated again that black tea was the most potent in inhibiting α-amylase and α-glucosidase among different teas (green, oolong and black teas) [65]. Black tea polyphenols have stronger inhibitory effects on α-amylase and glucosidases than green tea. In addition, black tea extract was demonstrated to inhibit the degradation of disaccharides into monosaccharides by α-glucosidase in the rat small intestine. The inhibition of disaccharide degradation prevented the absorption of glucose [66].

Dipeptidyl peptidase-4 (DPP-4) is a regulatory protease involved in the inactivation of a variety of proline-rich peptides, including glucagon line peptide-1 (GLP-1), neuropeptides, and other chemokines. GLP-1 is an insulinotropic hormone considered to be a therapeutic agent for the treatment of type 2 diabetes. It was also found that high-fat and high-cholesterol diets increased DPP-4 expression in intestinal lymph [67]. DPP-4 inhibition efficiently increased the active GLP-1 levels in *db*/*db* mice fed a diet containing sucrose and linoleic acid [68]. DPP-4 inhibition improved glucose tolerance, β cell function, and adipose tissue inflammation in db/db mice. Black tea was highly effective in inhibiting DPP-4 in vitro [69]. However, the effect of black tea on DPP-4 in vivo has not been investigated.

In a crossover study, it was demonstrated black tea extract induced malabsorption of 25% of the carbohydrates [21]. In an animal study, a sucrose-rich diet induced hypercholesterolemia and hypertriglyceridemia in rats. Consuming black tea extracts significantly decreased body weight gain and food efficiency. (In animal studies, food efficiency is the ratio of body weight gain to food intake. Body weight gain is calculated by subtracting the basal body weight from the body weight at the end of experiment. Food intake is the weight of the total food eaten by animals during the period of the experiment.) Black tea extracts showed lower food efficiency than green tea, which was consistent with the result of the inhibitory activity against α-amylase and glucosidases. Hypertriglyceridemia and hypercholesterolemia were normalized by black tea extract. The triglyceride content in the liver as well as the cholesterol content in the heart of rats fed a sucrose-rich diet were elevated and were normalized by black tea extract [70].

## 5. The Anti-Oxidant Effect of Black Tea Polyphenols

Oxidative stress participates in the pathological processes of obesity and the comorbidities of obesity (e.g., diabetes, arteriosclerosis). Many epidemiological studies revealed that obesity elevates systemic oxidative stress in humans [71]. The biomarkers of oxidative damage are higher in obese individuals and positively correlate with BMI, body fat rate, LDL oxidation, and TG levels [72]. Meanwhile, the antioxidant defense markers are negatively related to the weight of body fat and central obesity [73]. In turn, oxidative stress promotes the progression of obesity.

Black tea polyphenols are effective antioxidants. Theaflavins are able to activate glutathione peroxidase (GPx), glutathione-S-transferase (GST), catalase (CAT), and superoxide dismutase (SOD) in different degrees in mice [74]. Theaflavins were good antioxidants for scavenging reactive oxygen species (ROS) and preventing free radical–induced DNA damage in vitro [75]. The antioxidant properties of theaflavins were investigated by comparing them with EGCG [76]. It was found that TF1, theaflavin-3(3′)-gallate (TF2), and TF3 were more effective than EGCG in preventing H_2_O_2_-mediated damage in HPF-1 cells. Oxidized LDL (oxLDL) promoted the development of atherosclerosis. A study showed that four theaflavin derivatives exhibited a dose-dependent antioxidant activity in LDL oxidation at concentrations of 5–40 μmol/L [77]. Another study revealed that TF3 pretreatment of endothelial or macrophage cells dose-dependently and time-dependently reduced cell-mediated LDL oxidation by decreasing superoxide release from macrophages and chelating iron ions [78].

## 6. Conclusions

**Figure 1:**
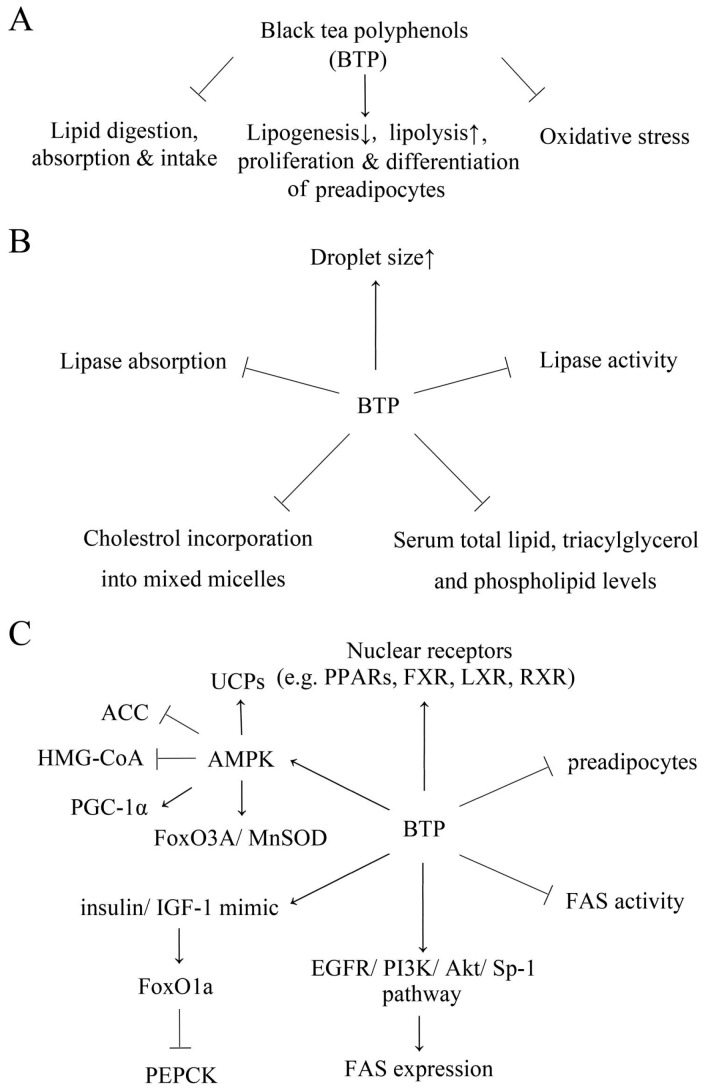
Anti-obesity mechanisms of black tea polyphenols. (**A**) General anti-obesity mechanisms of black tea polyphenols. Detailed anti-obesity mechanisms of black tea polyphenols include inhibiting lipid digestion, absorption and intake, thus reducing calorie intake (**B**); attenuating lipogenesis, enhancing lipolysis and decreasing the differentiation and proliferation of preadipocytes (**C**); and suppressing oxidative stress.

Black tea polyphenols have excellent anti-obesity activity without apparent side effects. A summary of the mechanisms by which black tea polyphenols act, including the inhibition of lipid and saccharide digestion, absorption and intake, promotion of lipid metabolism and blockage of the pathological processes of obesity and the comorbidities of obesity by reducing oxidative stress, is shown in Figure 1. Black tea polyphenols inhibit the emulsion of droplets and the activity of pancreatic lipase, α-amylase and glucosidases. Furthermore, black tea polyphenols may also decrease food intake to reduce energy intake. Black tea polyphenols attenuate lipogenesis and enhance lipolysis. AMPK and nuclear receptors play vital roles in the process. In addition, black tea polyphenols prevent the proliferation and differentiation of preadipocytes to decrease lipid accumulation.

However, compared with the investigation of the anti-obesity effect of green tea, more studies on black tea are needed. Green tea polyphenols could affect the gut microbiome in mice [79,80] which contributed to the anti-obesity effect of green tea polyphenols [79]. The possibility that black tea affects the gut microbiome was only studied in vitro [81] and the correlation between the anti-obesity effect of black tea and its effect on the gut microbiome has not been studied. The potential effects of polyphenols on food intake by influencing neuroregulatory factors, neural signaling pathways and/or peripheral feedback mechanisms have been studied [82]. Green tea polyphenols could reverse the effects of orexigenic genes in the obese rat [83]. The orexigenic effects of black tea polyphenols on the brain should be studied. Besides, epidemiological research on the relationship between obesity and black tea polyphenol consumption should be carried out to verify the effectiveness of black tea polyphenols on obesity.
